# Nanostraw membrane stamping for direct delivery of molecules into adhesive cells

**DOI:** 10.1038/s41598-019-43340-1

**Published:** 2019-05-02

**Authors:** Bowen Zhang, Yiming Shi, Daisuke Miyamoto, Koji Nakazawa, Takeo Miyake

**Affiliations:** 1 Graduate School of Information, Production and Systems, Waseda University, Kitakyushu Fukuoka, 808-0135 Japan; 2 Department of Life and Environment Engineering, The University of Kitakyushu, 1-1 Hibikino, Wakamatsu-ku, Kitakyushu Fukuoka, 808-0135 Japan

**Keywords:** Permeation and transport, Electrophysiology, Nanoscale materials

## Abstract

Delivering ions and molecules into living cells has become an important challenge in medical and biological fields. Conventional molecular delivery, however, has several issues such as physical and chemical damage to biological cells. Here, we present a method of directly delivering molecules into adhesive cells with an Au-based nanostraw membrane stamp that can physically inject a target molecule into the cytoplasm through a nanostraw duct. We successfully delivered calcein target molecules into adhesive cells with high efficiency (85%) and viability (90%). Furthermore, we modeled the molecular flow through Au nanostraws and then demonstrated the control of calcein flow by changing the concentration and geometry of Au nanostraws. Our Au membrane stamping provides a new way of accessing the cytoplasm to modulate cellular functions via injected molecules.

## Introduction

Gaining access to the cytoplasm of cells is now a significant challenge in biological research to analyze and modulate cellular functions^1^. Examples include gene injection to create induced pluripotent stem (iPS) cells^2^ or the inhibition of gene expression inside cells, which is called as RNA interference^3^.

**Figure 1:**
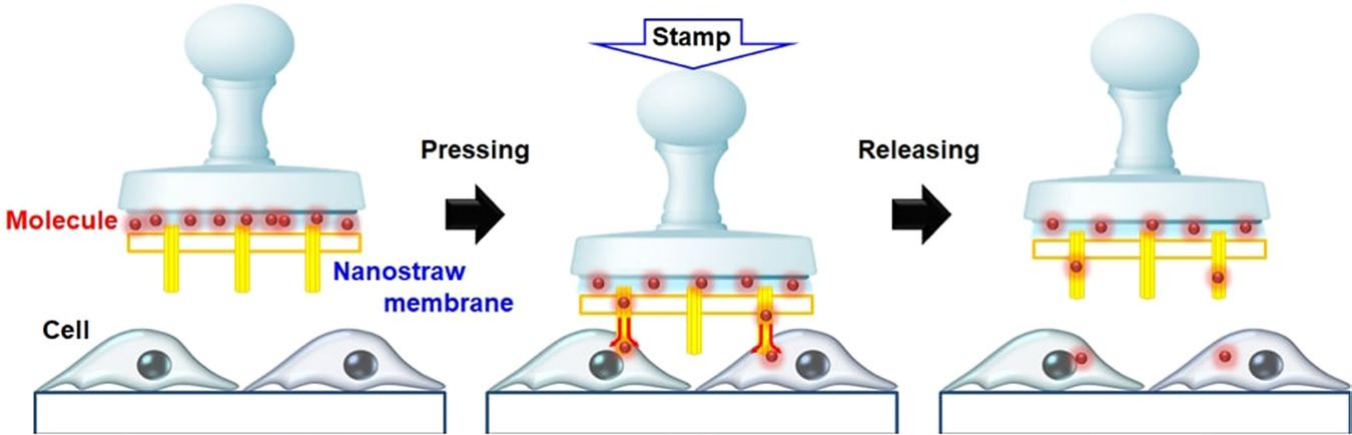
Nanostraw membrane stamping for direct intracellular molecular delivery.

A key challenge for intracellular access is to overcome the physical barrier of the outer membrane of cells without losing cellular functions^4^. Such research efforts have been classified as follows: (1) carrier-based delivery using viral vectors^5^, vesicles^6^, and nanocarriers including lipids^7^ and inorganic materials^8^ and (2) membrane-disruption delivery via mechanical^9,10^, electrical^11^, thermal^12^, and optical^13^ means. Each of these methods has specific advantages and disadvantages in terms of efficiency, invasiveness, safety, dosage control, and cost^4^. Alternatively, direct delivery using a solid nanoconduit is a noteworthy technique to deliver/extract molecules into/from living cells^14^. Carbon nanotube-^15^ and glass nanopipette-based endoscopes^16^ have also provided methods for minimally invasive insertion into cells, and controlled the flow of delivery and extraction of Ca^2+^ ^17^, proteins^2^, and mRNAs^18^. Examples of this using many nanomaterials have been reported, including vertical Si nanowires^19^, Si nanopillars^20^, Si_3_N_4_^11,21^, and Au nanoelectrode^9^. Moreover, aluminum nanostraws^14^, which are arranged in a polycarbonate membrane, have been used as a powerful tool for delivering therapeutic DNA^22^ or drugs^23^ with improved transfection efficiency. However, even with the use of specific nanomaterials, molecular delivery into cells is difficult to control; therefore, this approach has been combined with other techniques such as electroporation^24^, optical stimulation^25^, and microfluidics^14^. In addition to these recent efforts focusing on molecular delivery using specific nanomaterials combination methods, we have developed a method of mechanical Au nanostraw membrane stamping with a height-adjustable stage controller (Fig. 1). Here, we investigate the optimal diameter and length of such Au nanostraws to penetrate and inject molecules into cells, and also evaluate the rate of delivery into adhesive cells and cellular viability. Furthermore we demonstrate the delivery using two types of molecules (small molecule: calcein dye (622.55 MW) and large molecule: fluorescein (FAM) labeled oligo DNA (6603.51 MW)) into two types of adhesive cells (NIH3T3 fibroblast and HeLa cancer cells) with high efficiency and viability. This is a simple and novel method compared with prior works using aluminum nanostraws^26^, Si^27^ and carbon nanotube^28^ nanomaterials that allows for direct molecular delivery through the nanostraws inserted mechanically into the cellular membrane.

## Results

### Au nanostraw membrane stamp

**Figure 2:**
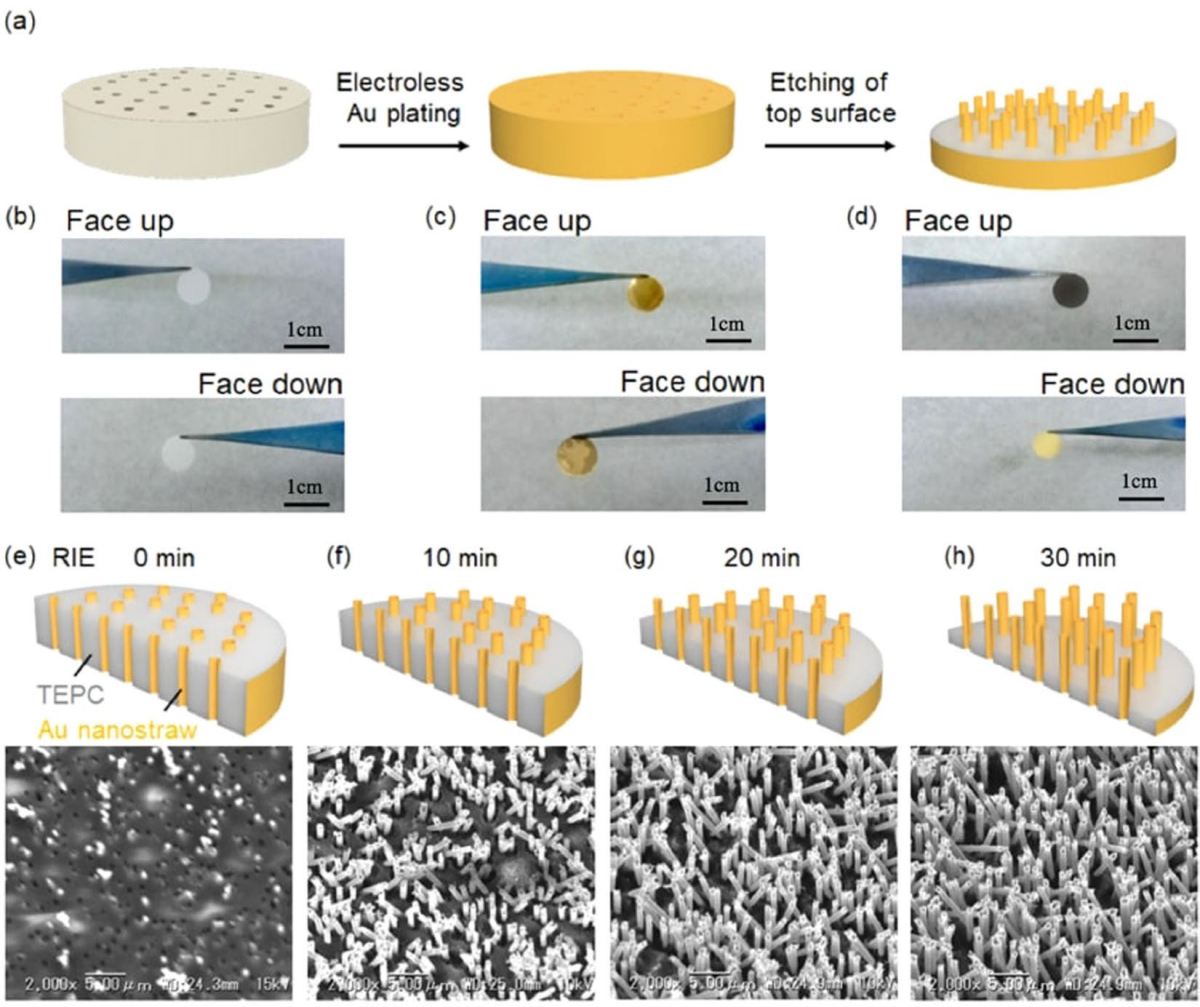
Nanostraw membrane fabrication. (**a**) Two steps for the nanostraw fabrication: (1) electroless Au plating to track-etched polycarbonate (TEPC) membrane and (2) etching of top surface. (**b–d**) Optical images of 8-mm-diameter samples at different stages of nanostraw fabrication. TEPC membrane included size-controlled pores. In our experiments, 0.4-, 0.6-, and 1.0-μm-diameter pores were used. (**b**) Images of the original TEPC membrane facing up and down. (**c**) Au is plated on the TEPC membrane surfaces as well as the pore surfaces. (**d**) Top surface of Au membrane was etched by two steps: (1) Au surface etching with aqua regia, including nitric acid and hydrochloric acid, and (2) PC surface etching with O_2_ RIE. (**e–h**) The Au/TEPC nanostraws after RIE at 0 (**e**), 10 (**f**), 20 (**g**), and 30 min (**h**).

To make Au nanostraw membranes, we investigated electroless Au plating on track-etched polycarbonate (TEPC) templates and subsequent etching of the top surface (Fig. 2a). Electroless deposition of an Au nanolayer onto a porous membrane is a well-established plating process that consists of four steps: (1) sensitization, (2) activation, (3) displacement deposition, and (4) electroless deposition (Fig. S1a). To activate the surface of TEPC membranes (pore diameter: 600 nm, thickness: 23 µm), we immersed the TEPC membrane in 10.55 mM SnCl_2_ solution at 24 °C and then in 11.28 mM PdCl_2_ solution at 24 °C. After washing the membrane with water, the tin–palladium metallic layer was formed on the TEPC surface, and could be used as a catalyst for electroless gold plating. The amount of metallic catalyst on the TEPC surface was controlled by adjusting the immersion time and the number of activation cycles (Fig. S1b). After that, we immersed the catalyst-coated TEPC membrane in 2 g/L gold plating solution at 40 °C for 24 h. After coating Au nanolayer on the membrane, the original white color of the TEPC membrane (Fig. 2b) changed to gold on both the top and the bottom surfaces (Fig. 2c). Furthermore, we investigated the cross section of Au/TEPC membranes using a scanning electron microscope (SEM) to confirm that Au intrananotubes had formed after an immersion time of 5 min for one to four cycles (Fig. S1c-f, respectively). We confirmed the presence of Au intrananotubes in the TEPC membrane after more than three cycles (Fig. S1e,f) due to a sufficient metallic catalyst coating and subsequent gold plating onto the TEPC pore surface. When we performed immersion in SnCl_2_ and PdCl_2_ solutions for a longer time (10 and 20 min), we successfully formed Au intrananotubes upon treatment for two cycles. Eventually, we created a process map for electroless Au plating onto 600-nm-pore TEPC membranes (Fig. S1b).

After forming the Au/TEPC membrane, we performed etching of only the top surface of the Au deposition nanolayer with aqua regia, which was mixed with nitric acid and hydrochloric acid at a molar ratio of 1:3, to expose both the polycarbonate and the internal Au nanoducts on the membrane surface (Fig. 2a,e). After wet etching, we observed a change of color from gold to brown only on the etched surface (Fig. 2d). Furthermore, we confirmed the outer and inner diameters of the exposed Au nanotubes (${\varnothing }_{outer}$ = 600 nm ± 90 nm, ${\varnothing }_{inner}$ = 400 nm ± 50 nm, needle density n = 4 × 10^7^) using SEM (Fig. 2e). We summarize the geometry of Au nanotubes on TEPC membranes with different pore sizes (400, 600, and 1000 nm) in Table S1. From these data, a 100 ± 30 nm Au layer was formed on the TEPC membrane upon immersion in gold plating solution for 24 h. Additional etching of exposed TEPC with O_2_ plasma produces hollow Au nanoneedles, which we refer to as nanostraws. The height (H) of Au nanostraws was controlled by adjusting the O_2_ plasma exposure time: as 0 min (Fig. 2e, H: 0 µm), 10 min (Fig. 2f, H: 1.3 µm), 20 min (Fig. 2g, H: 2.4 µm), or 30 min (Fig. 2h, H: 5.0 µm). Since the exposed TEPC membrane was damaged by activated O_2_ molecules in plasma, we could not perform O_2_ exposure for longer than 40 min to make free-standing Au nanostraws/TEPC membranes.

### Molecular injection into cells

**Figure 3:**
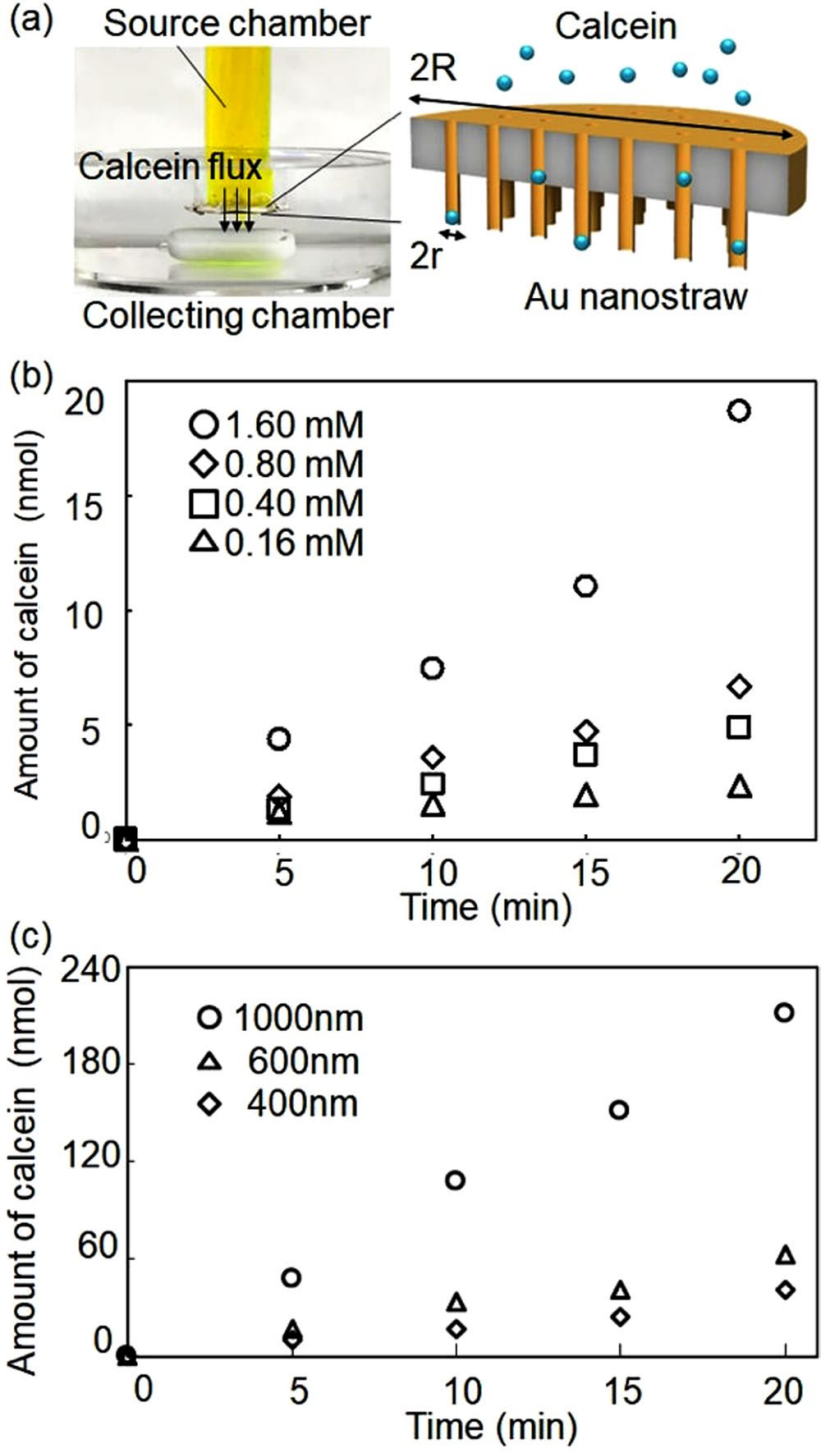
Calcein flow through Au nanostraw/TEPC membrane. (**a**) Photograph and schematic of calcein flow from the source chamber to the target chamber via Au nanostraws/TEPC membrane. (**b**) Amount of transported calcein at the different concentrations of calcein in the source chamber. (**c**) Amount of transported calcein with the different diameter Au nanostraws/TEPC at 1.6 mM calcein concentration.

To calibrate the molecular flow through the Au nanostraw/TEPC membrane, we first investigated the injection flux of calcein molecules from a calcein-containing source chamber to the target chamber of the stirred phosphate buffer solution (PBS) (Fig. 3a). We made a needle-type source chamber consisting of an 8-mm-diameter membrane including Au nanostraws (outer diameter: 400 ± 50 nm, inner diameter *r*: 200 ± 30 nm), a glass tube (outer diameter: 8 mm, inner diameter *R*: 6 mm), and 10 mM PBS (pH 7) including target calcein molecules at different concentrations. When we contacted the needle source chamber with the stirring PBS solution in the collection target chamber, the calcein diffused from a high concentration in the source chamber to the target chamber via the Au nanostraw duct. We measured the amount of transported calcein in the target chamber using a microplate reader. This calcein flux *J* through the nanostraw membrane is defined as follows^29^:

1 $$J=\frac{DC(\pi {r}^{2}n/\pi {R}^{2})}{l}$$

where *D* is the diffusion coefficient, *C* is the calcein concentration, *πr*^2^ is the inner area of Au nanostraw, *n* is number of Au nanostraws on membrane area (*πR*^2^), *πR*^2^ is the membrane area, and *l* is the thickness of the membrane.

According to equation (1), the calcein flows through the Au nanostraw membrane as a function of *C* and *πr*^2^. When we used the Au nanostraws (400-nm diameter)/TEPC membrane with 0.16 mM calcein solution, the amount of transported calcein in the target chamber increased linearly up to 2.3 nmol for 20 min (Fig. 3b), resulting in calcein flux *J* of 0.3 nmol s^−1^ cm^−2^. *J* was enhanced to 0.7 nmol s^−1^ cm^−2^ by increasing the calcein concentration to 0.4 mM, 1.1 nmol s^−1^ cm^−2^ when increasing it to 0.8 mM, and 2.3 nmol s^−1^ cm^−2^ when increasing it to 1.6 mM. Further improvement was achieved by modifying the inner area of nanostraws (Fig. 3c). *J* was 1.4 times greater (3.0 nmol s^−1^ cm^−2^) with Au nanostraws (600-nm diameter)/TEPC membrane, and 2.3 times greater (5.1 nmol s^−1^ cm^−2^) with Au nanostraws (1000-nm diameter)/TEPC membrane than the *J* obtained with the Au nanostraws (400-nm diameter)/TEPC membrane. These results indicate that the amount of delivered calcein target molecules can be controlled by adjusting *C* and *πr*^2^.

**Figure 4:**
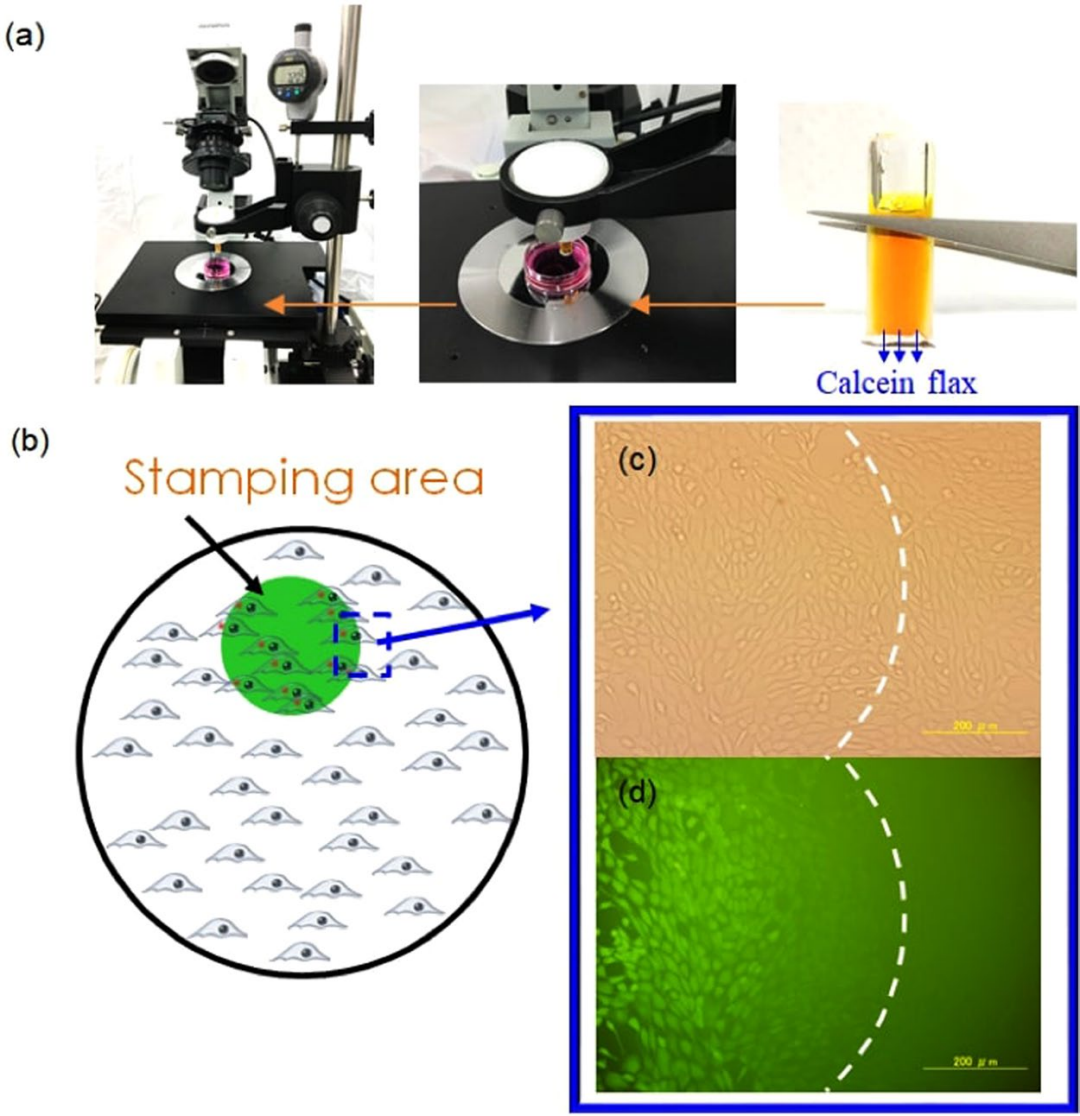
Calcein delivery into NIH-3T3 cells with Au nanostraw stamping. (**a**) Photograph of Au nanostraw stamping system for precise x-y-z manipulation. (**b**) Schematic illustration of stamping area using Au nanostraw membrane on the cell-cultured dish. (**c,d**) Optical (**c**) and fluorescence (**d**) images at the edge of stamped and unstamped areas.

Similar to micro/nanoneedle intracellular delivery systems, our Au nanostraw membrane injection system requires precise x-y-z manipulation. To this end, we combined an optical microscope, including an x-y stage with an Au nanostraw membrane stamp moving manually in the z-axis with 1-µm step accuracy (Fig. 4a). The Au nanostraw membrane stamp consists of an 8-mm-diameter membrane including Au nanostraws with a 600-nm outer diameter and different heights (0, 1.3, 2.4, and 5.0 µm), a glass tube (outer diameter: 8 mm, inner diameter: 6 mm), and 1.6 mM calcein dye in 10 mM PBS at pH 7. As an adhesive cell, we used NIH-3T3 fibroblast cells cultured on a plastic Petri dish filled with Dulbecco’s modified Eagle medium at 37 °C in 5% CO_2_ for 2–3 days. Before calcein injection, we confirmed 80–100% confluency of the NIH-3T3 cells with a confocal microscope and then stamped the Au nanostraw membrane including calcein dye onto adhesive cells (Fig. 4b). After the stamping, we observed optical (Fig. 4c) and fluorescence (Fig. 4d) images focused on the region where stamped and unstamped areas met. From these images, we clearly confirmed the presence of stained cells only in the stamped area. In control experiments of the cultured cells exposed to 1.6 mM calcein medium for 10 min (Fig. S2), we did not observe any stained cells. These results indicate that calcein molecules can be delivered through Au nanostraws.

**Figure 5:**
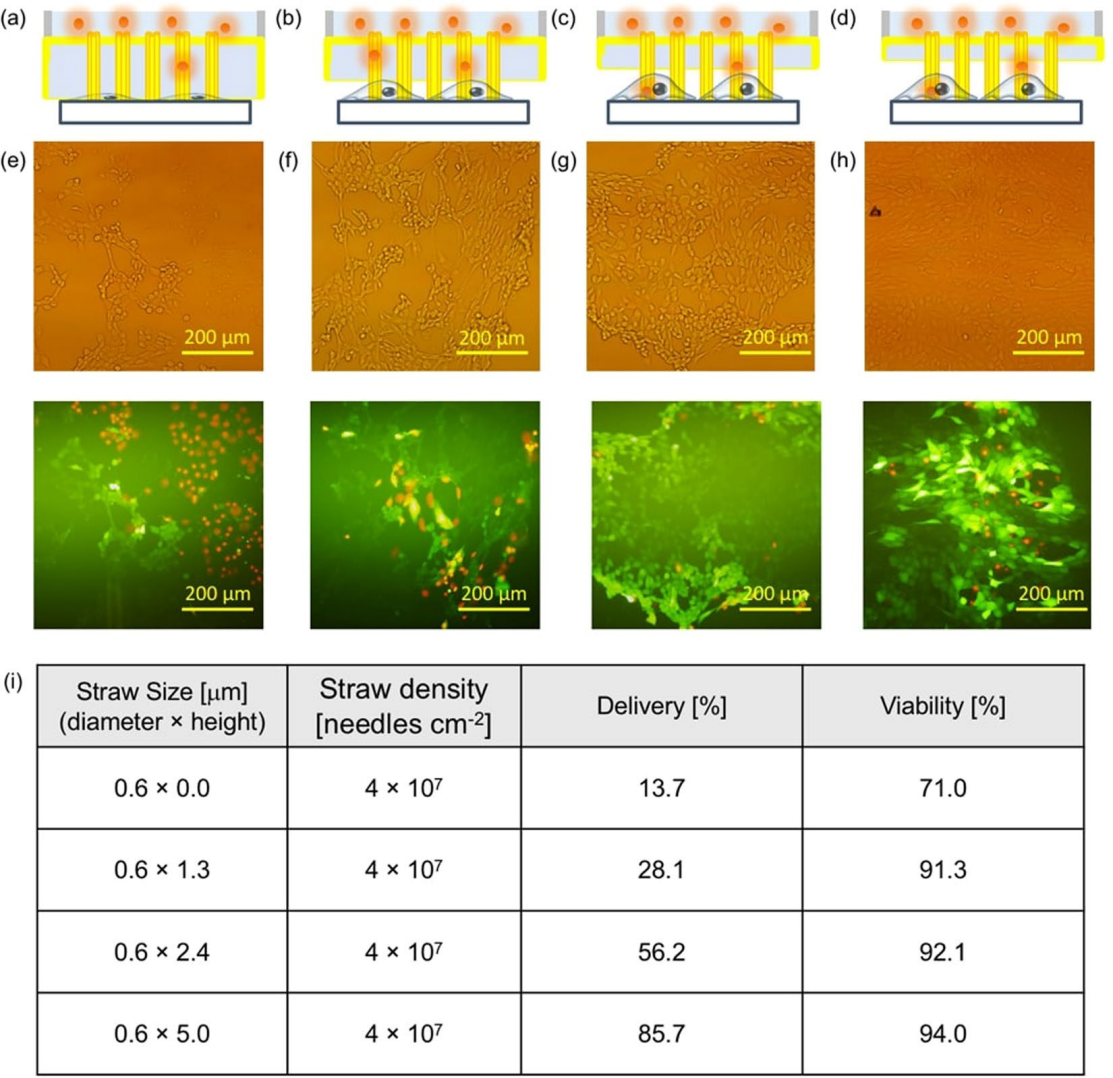
Direct delivery of molecules into NIH-3T3 cells. (**a–d**) Schematics of Au nanostraw membrane stamping into cells with different nanostraw heights of 0 (**a**), 1.3 (**b**), 2.4 (**c**), and 5.0 µm (**d**). (**e–h**) Optical and fluorescence images after the calcein and PI delivery using 0- (**e**), 1.3- (**f**), 2.4- (**g**), and 5.0-µm-high nanostraws (**h**). Total cell number N = 224 in (**e**), 476 in (**f**), 535 in (**g**) and 494 in (**h**). (**i**) Summarized data of nanostraw size and density, molecular delivery, and viability.

To confirm the feasibility of intracellular molecular delivery using Au nanostraw stamping, we investigated the viability (%) and delivery (%) after stamping with Au nanostraw membranes of different heights (Fig. 5). The viability and the delivery are defined as follows: *Viability* = *live cells/live and dead cells* and *Delivery* = *live cells/total cells*, where live cells are the number of calcein-stained cells, dead cells are the number of propidium iodide (PI)-stained cells, and total cells are the number of optically observed cells. Since calcein is a membrane-impermeable dye, the cells were not stained in calcein-containing medium (Fig. S2). Instead, we could inject a calcein dye into the cells through the Au nanostraw duct. In general, such Au nanostraw stamping leads to the generation of insertion holes in the cellular membrane that are repaired rapidly in healthy cells, resulting in the injected calcein being trapped inside the cells, which we refer to as live cells. In contrast, when the cells are damaged, the membrane holes remain until cell death, resulting in the binding of PI to intracellular DNA, which we refer to as dead cells. After stamping with Au nanostraws, the stamped cells retained high viability of over 90% with Au nanostraws of different heights of 1.3, 2.4, and 5.0 µm (Fig. 5i). However, the delivery rate varied from low at 13.7% with 0-µm-high nanostraws to high at 85.7% with 5.0-µm-high nanostraws. This is because higher Au nanostraws can penetrate easily into the adsorbed NIH-3T3 fibroblast cells, the height of which is less than 4 µm at the center^30^ and less than 0.8 µm at the edge^31^. Furthermore, we demonstrated the delivery of molecules into NIH-3T3 cells with different nanostraw diameters of 0.4 and 1.0 µm, at a height of 5.0 µm (Fig. S3). We confirmed similar results of 90.1% viability/75.7% delivery using 400-nm-diameter Au nanostraw and 92.6% viability/87.2% delivery using 1000-nm-diameter Au nanostraw membrane, when compared with the data using 600-nm-diameter nanostraws. Our nanostraw stamping technique allows the delivery of calcein and large molecule (FAM-labeled oligo DNA) into the different types of the cell (HeLa cancer cell) (Fig. S4). We confirmed the results of 94.6% viability/87.2% delivery with small calcein and 90.3% viability/83.0% delivery with oligo DNA, so our nanostraw stamping system provide a versatile method for molecular delivery into the different types of adhesive cells.

## Discussion and Conclusions

We have demonstrated the direct delivery of molecules into adhesive NIH-3T3 cells with Au nanostraw membrane stamping, resulting in high cell viability of over 90% and highly efficient delivery of 85%. Such high viability and efficient delivery were achieved by the modification of Au nanostraw insertion geometry. The flow rate of calcein molecules at the Au nanostraws was controlled by adjusting the calcein concentration in the source chamber and the inner diameter of the Au nanostraws. These improvements reflect two critical advantages of using Au nanostraw membrane for intracellular molecular delivery. First, Au nanostraws provide sufficient mechanical properties to penetrate the cell membrane and to minimize the damage to cells during the molecular delivery. Second, with the performance of stamping a single time, we can inject the target molecules into multiple adhesive cells. This is the first demonstration of the use of Au nanostraw membranes for intracellular molecular delivery. In future applications, our nanostraw stamping system could be applied to deliver functional molecules of mRNA to edit and manipulate cellular functions^24^, as well as peptides and proteins^32^ to modulate and characterize cellular signaling pathways, and also could be used to intracellular extraction^33^ and medicine delivery^34^.

## Materials and Methods

### Electroless Au thin-film deposition

The TEPC membranes (it4ip S.A.) were treated in 1.25 M NaOH solution for 20 min at 40 °C. After washing the membranes with water, they were immersed in 1.25 M SnCl_2_ solution for 10 min at 25 °C and then washed with water. Sn^2+^-coated TEPC membrane was immersed in 1.25 M PdCl_2_ solution for 10 min at 25 °C to form the metallic catalysts of Sn^4+^-Pd on the membrane surface. We repeated this metallic formation cycle one to four times. The catalyst-coated TEPC membrane was immersed in an electroless gold planting solution including 200 ml/L NC gold PDII (NC Gold II; Kojima Chemicals) and 20 ml/L gold(1) trisodium disulphite, for 24 h at 40 °C. After washing the Au/TEPC membrane with water, we dried it in a vacuum chamber.

### Au nanostraw fabrication with wet and dry etching

An Au/TEPC membrane was floated on aqua regia (ITO-02; Kanto Chemical Co., Inc.) for 4 min to etch the top surface of the Au nanolayer on the Au/TEPC membrane. After washing the etched Au/TEPC membrane with distilled water, it was dried in a vacuum chamber. To make Au nanostraws, the TEPC surface on the Au/TEPC membrane was etched with oxygen-based reactive ion etching. By changing the etching time, the height of the Au nanostraws could be controlled. After etching, scanning electron microscopy (SEM) images of Au nanostraws were obtained using a HITACHI SEM S-3400N, and use HITACHI SEM software to measure the outer and inner diameters of more than 100 Au nanostraws.

### Cell culture

We used NIH 3T3 cells (JCRB0615; Health Science Research Resources Bank, Japan Health Science Foundation, Japan) and HeLa cells (JCRB9004; Health Science Research Resources Bank, Japan Health Science Foundation, Japan) as adhesive cells. The cells were cultured in Dulbecco’s minimal essential medium (Invitrogen Corp., USA) supplemented with 10% fetal bovine serum, 100 U mL^−1^ penicillin, and 100 µg mL^−1^ streptomycin. Each cell suspension was obtained by treating the confluent monolayer formed on the tissue culture dish with 0.25% trypsin (Invitrogen). The cells were cultured under a humidified atmosphere of 5% CO_2_ and 95% air at 37 °C.

### Intracellular molecular delivery

We made a needle-type source chamber consisting of Au nanostraws/TEPC membrane (8-mm diameter), a glass tube (outer diameter: 8 mm, inner diameter *R*: 6 mm), and 10 mM PBS (pH 7), including target molecules at different concentrations. Typically we used 1.6 mM calcein (MP Biomedicals, Inc., MW: 622.5) and 0.1 mM oligo DNA (FAM-labeled TTTTATTTTGTTTTCTTTTG, BED.CO., MW: 6603.51) solution. A needle-type source chamber was set in a stand that moved along the z-axis at 1-µm steps and then combined it with an optical microscope (Olympus IX71), including an x-y stage, to establish a precise x-y-z manipulation system. By using this manipulator, we inserted Au nanostraws into adhesive NIH-3T3 cells and then delivered the calcein molecules into the cells through Au nanostraw ducts for 1 min. After exchanging the medium solution, we observed the calcein-stained cells with a fluorescent microscope (Olympus IX71). To confirm the dead cells, we injected 0.05 ml of PI at 4 μM into a 10-ml cell-culture dish and then performed culturing for 30 min. After exchanging the medium solution, we counted the PI-stained cells in the fluorescent images.

## Supplementary information

Supplemental data
