# Supplementary material for: Nanostraw membrane stamping for direct delivery of molecules into adhesive cells
Source: Sci Rep. 2019 May 2;9:6806. doi: 10.1038/s41598-019-43340-1 (PMC6497648; doi:10.1038/s41598-019-43340-1)
Supplement: Supplementary file 1 — Supplemental data [file 41598_2019_43340_MOESM1_ESM.docx]

**Supplementary Materials**

**Nanostraw membrane stamping for direct delivery of molecules into adhesive cells**

Bowen Zhang,^1^ Yiming Shi,^1^ Daisuke Miyamoto,^2^ Koji Nakazawa,^2^ Takeo Miyake^1,*^

^1^Graduate School of Information, Production and Systems, Waseda University, Kitakyushu, Fukuoka 808-0135, Japan

^2^Department of Life and Environment Engineering, The University of Kitakyushu, 1-1 Hibikino, Wakamatsu-ku, Kitakyushu, Fukuoka 808-0135, Japan

^*^Corresponding author: miyake@waseda.jp


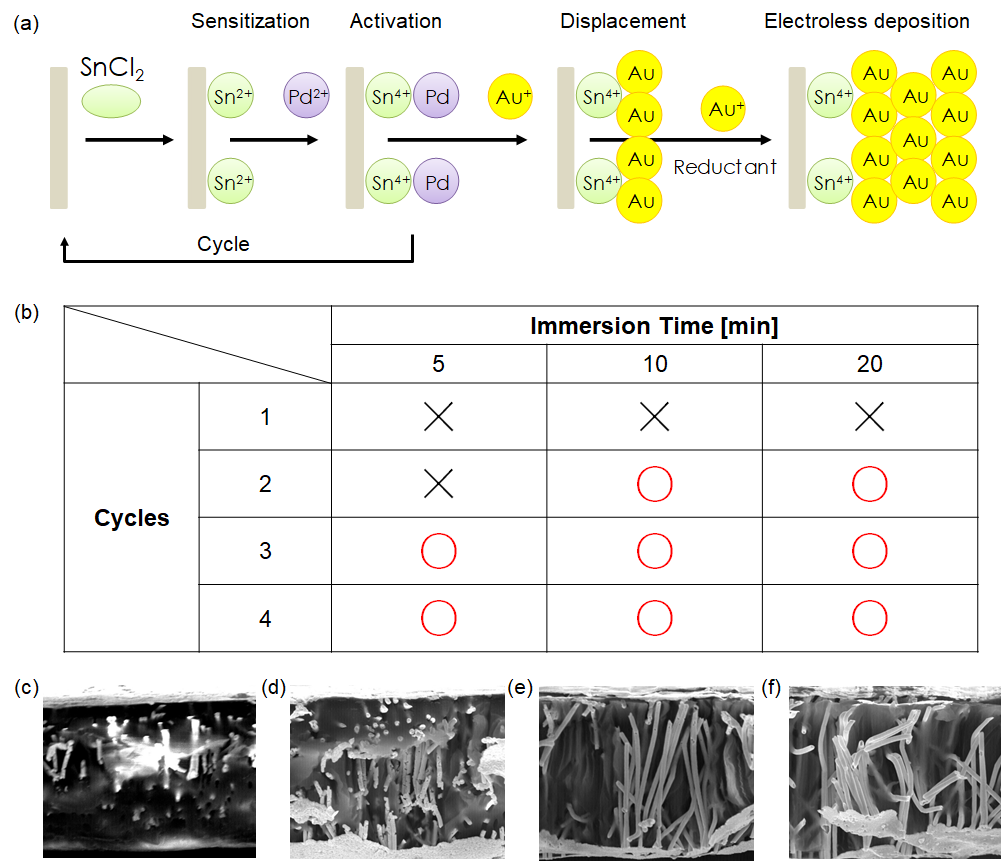


**Figure S1.** (a) Process flow for fabricating Au nanostraws on polycarbonate membrane. (b) Process map of Au nanostraw membrane. (c–f) Cross-sectional SEM images of Au nanostraw membrane for 5-min immersion time at the different numbers of cycles of sensitization and activation.

**Table S1. Outer and inner diameters of Au nanostraws on the templates with different pore sizes.**

**
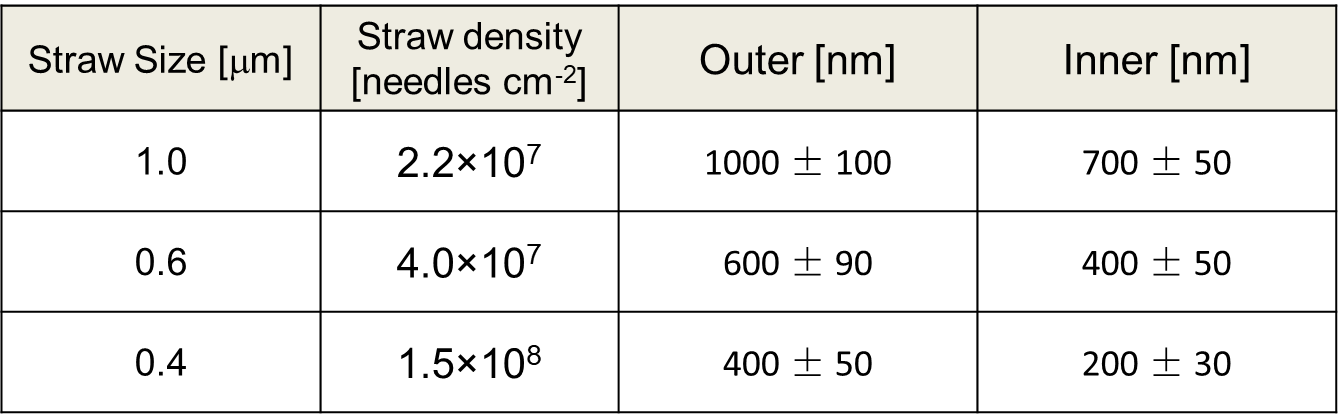
**


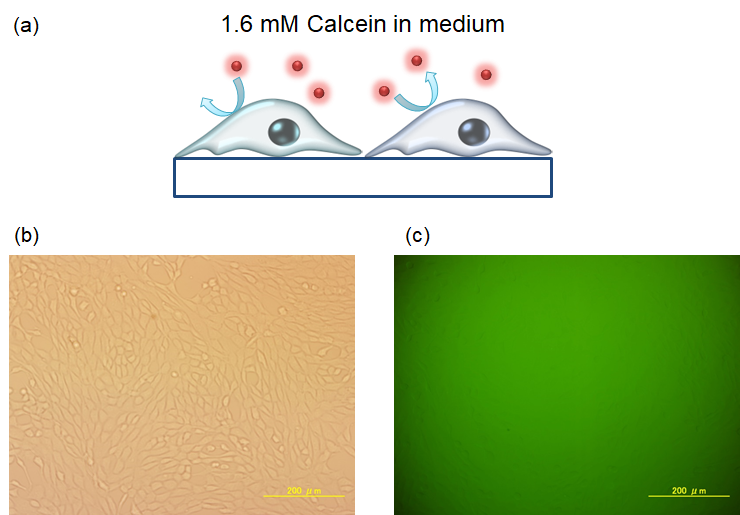


**Figure S2.** (a–c) The cells remained unstained upon exposure to medium including 1.6 mM calcein. (b) Optical image of unstained NIH-3T3 cells cultured on a dish. (c) Fluorescence image of calcein-exposed NIH-3T3 cells.

**
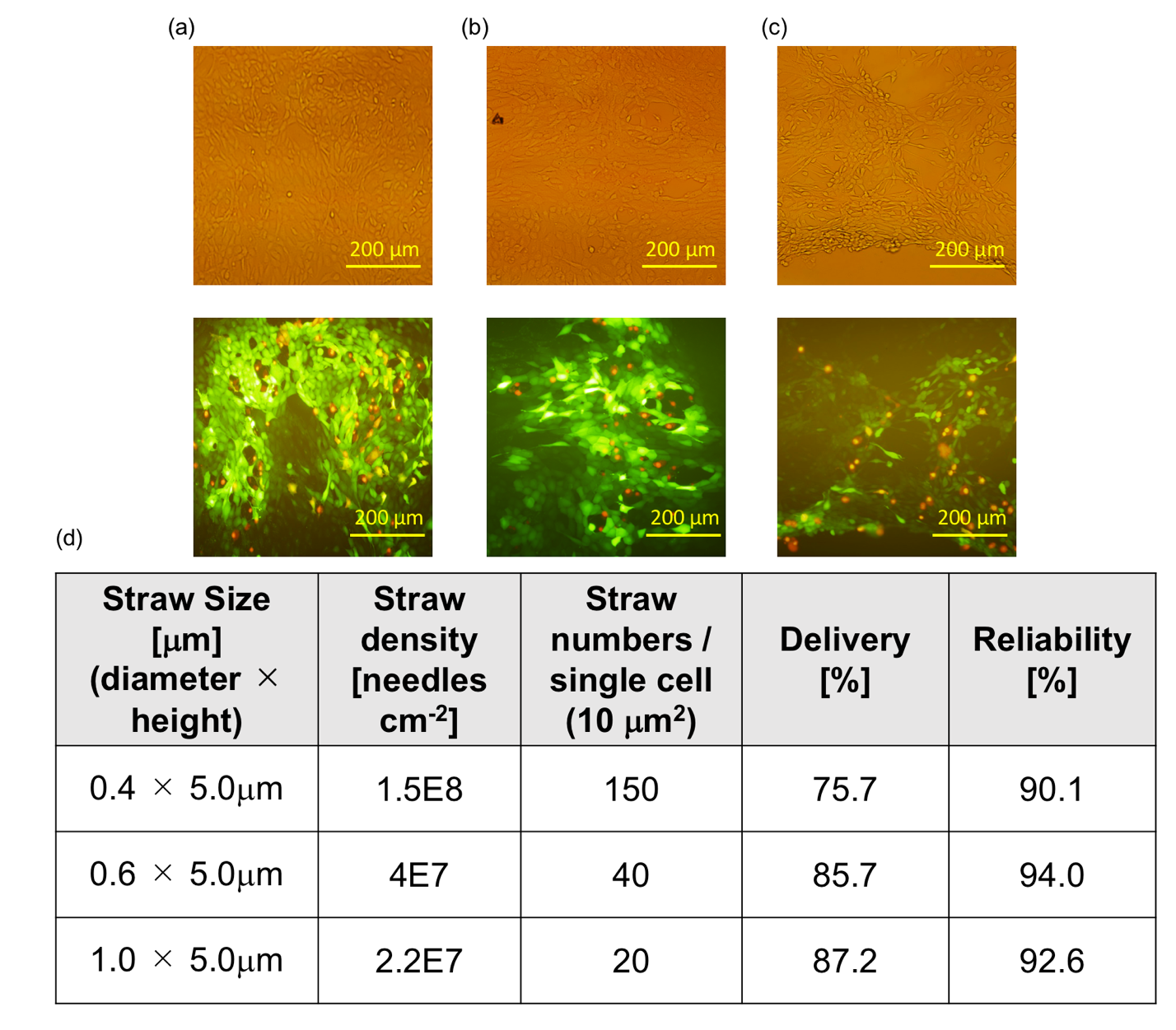
**

**Figure S3.** (a–c) Optical and fluorescence images after the calcein and PI delivery using 0.4- (a), 0.6- (b), and 1.0-µm-diameter nanostraws (c). Total cell number N = 529 in (a), 494 in (b), 548 in (c). (d) Summarized data of nanostraw size and density, molecular delivery, and viability

**
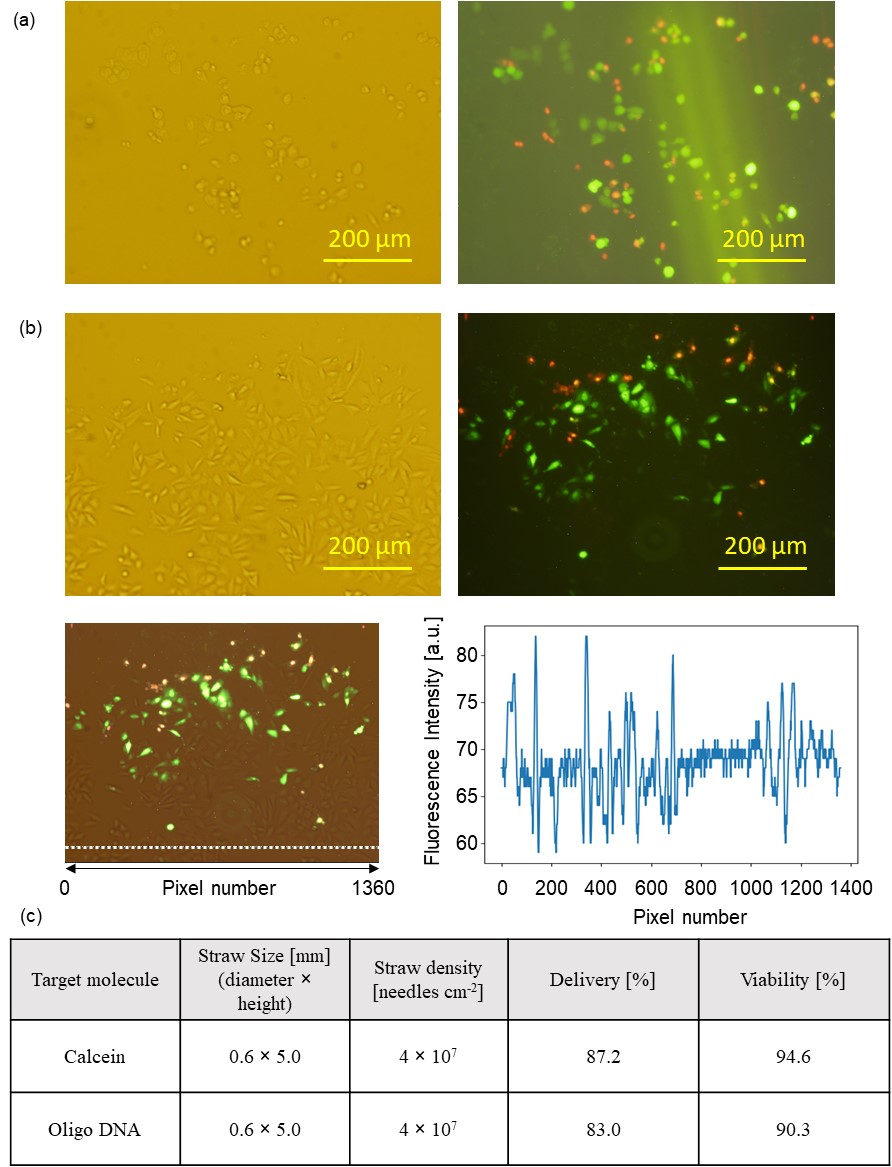
**

**Figure S4. Direct delivery of molecules (Calcein and FAM-labeled oligo DNA) into HeLa cells.** (a) Optical and fluorescence images after the calcein and PI delivery (b) Optical and fluorescence images after the FAM-labeled DNA and PI delivery and intensity profile of the image region marked by the dashed line. Fluorescence intensity from the stained cells (over 74) is higher than average background intensity (67). (c) Summarized data of nanostraw size and density, molecular delivery, and viability. N = 251 for calcein delivery and 371 for DNA delivery.
